# The Long-Term Functional Effect of Thrombectomy on Patients with Middle Cerebral Artery Occlusion Who Exhibit Moderate to Severe Disability

**DOI:** 10.3390/medicina57050509

**Published:** 2021-05-19

**Authors:** Ho-Jun Yi, Dong-Hoon Lee, Bo-Young Hong, Seung-Yoon Song, Yeun-Jie Yoo, Mi-Jeong Yoon, Jae-Hoon Sung, Seong-Hoon Lim

**Affiliations:** 1 Department of Neurosurgery, Soonchunhyang University Bucheon Hospital, Bucheon 14584, Korea; 431anarchy@naver.com; 2 Cerebrovascular & Endovascular Center, Department of Neurosurgery, St. Vincent’s Hospital, College of Medicine, The Catholic University of Korea, Suwon 16247, Korea; hoonydong@naver.com (D.-H.L.); shforl86@naver.com (S.-Y.S.); 3 Department of Rehabilitation Medicine, St. Vincent’s Hospital, College of Medicine, The Catholic University of Korea, Suwon 16247, Korea; byhong@catholic.ac.kr (B.-Y.H.); nugry@naver.com (Y.-J.Y.); allogen@naver.com (M.-J.Y.)

**Keywords:** stroke, endovascular thrombectomy, middle cerebral artery, recovery, outcome, Barthel Index, gait

## Abstract

*Background and Objectives*: Endovascular thrombectomy (EVT is an emerging gold standard treatment for acute cerebral infarction and may allow functional improvement after subacute cerebral infarction. However, the long-term functional benefits of EVT in patients with moderate to severe disability remain unclear. We investigated the effects of EVT on the activities of daily living (ADL), handicap, gait, and eating in patients with middle cerebral artery (MCA) occlusion who exhibited moderate to severe disability (score of 3–5 on the modified Rankin scale (mRS)) due to stroke, up to six months after onset. *Materials and Methods*: This retrospective longitudinal case–control study assessed 45 patients with MCA occlusion who exhibited moderate to severe disability (mRS score ≥ 3): 15 underwent EVT and 30 served as controls. Clinical assessments were conducted at two weeks (12–16 days), four weeks (26–30 days), and six months (180–210 days) after stroke onset. Functional assessments comprised the Korean version of the modified Barthel index (MBI), mRS, functional ambulation category (FAC), and dysphagia outcome severity scale (DOSS) to assess disability, handicap, gait, and eating. *Results*: The MBI, mRS, FAC, and DOSS scores all improved significantly (all *p* < 0.05) in the EVT group, compared to the controls. *Conclusions*: EVT has favorable effects on performing routine ADL, the handicap itself, walking, and eating. Therefore, EVT is recommended for patients with acute MCA occlusion, including those with severe disability at the initial assessment.

## 1. Importance

Endovascular thrombectomy (EVT) is an emerging gold standard treatment for acute cerebral infarction. Among the patients with MCA occlusion who exhibited moderate to severe disability, EVT had favorable effects on disability, handicap, gait, and eating function up to six months after onset.

## 2. Introduction

Functional recovery and prognosis are important considerations with patients with stroke. Functional recovery of the activities of daily living (ADL) may continue for up to six months after stroke onset [1,2,3]. The National Institutes of Health Stroke Scale scores, early nutritional status, and vascular supply (determined using susceptibility-weighted imaging) can all be evaluated to determine a patient’s acute prognosis after stroke [4,5]. Lesion location and white matter integrity also affect prognosis [6,7,8,9].

Endovascular thrombectomy (EVT) is an emerging gold standard treatment for acute cerebral infarction, currently [10,11]. Numerous randomized controlled trials (RCTs) have indicated that mechanical thrombectomy with retrievable stent or balloon-guided catheter, represents an effective therapeutic procedure in patients with large artery occlusion [10,12,13,14]. EVT improves functional recovery at three months after onset [11,15]. However, few studies of EVT have examined its functional benefits in patients with cerebral infarction who exhibit moderate to severe disability (i.e., those with a modified Rankin scale (mRS) score worse than 2).

We hypothesized that EVT might lead to favorable long-term functional outcomes in patients with middle cerebral artery (MCA) occlusion who exhibit even moderate to severe disability caused by severe stroke. Therefore, we investigated the effects of EVT on ADL, handicap, gait, and eating in patients with MCA infarcts and moderate to severe disability (mRS score = 3–5), all of whom were followed for six months after onset.

## 3. Material and Methods

### 3.1. Study Design and Participants

This retrospective longitudinal case–control study included 45 patients with first-ever stroke from a single center between January 2013 and December 2019. Thereof, 15 patients underwent EVT and 30 served as controls. All patients had unilateral MCA occlusion and met the following criteria: (1) 20–89 years of age; (2) first-ever stroke diagnosed by computed tomography (CT) or magnetic resonance imaging (MRI); (3) mRS scores ≥ 3 based on stroke at baseline and within 2 weeks after onset; and (4) initial assessment within 2 weeks after onset, subsequent assessment at 4 weeks after onset, and final assessment at approximately 6 months after onset. Exclusion criteria were as follows: (1) any other brain disorders other than stroke and (2) any other medical disorder resulting in non-independent gait and ADL. The patients were divided into a group ‘Thrombectomy’ who underwent EVT for unilateral MCA occlusion and achieved re-canalization (*n* = 15) and the other group ‘Control’ who with same unilateral MCA occlusion lacked the indications for EVT and had conservative treatment (*n* = 30).

All rehabilitation programs began within 5 days after onset. Treatment continued until 6 months after onset and consisted of both physical (neurodevelopmental treatment approach) and occupational (task-orientated approach) therapy for 1–2 h per day, 5 days per week [9,16]. Speech therapy was provided as needed.

The present study was a retrospective longitudinal study, so the exact sample size was not calculated beforehand. In addition, a long-term longitudinal study for patients with or without EVT for unilateral MCA occlusion has not been investigated yet. In recruiting period, many patients did not match our inclusion criteria (1) unilateral MCA occlusion, (2) cut-off values of mRS scores ≥ 3, and (3) evaluated up to 6 months after onset. In total, 15 patients were finally enrolled. For stroke control, to increase the accuracy of our results, we decided to recruit the stroke control group twice numbers of the EVT group. We tried to match the gender and age in all patients with unilateral MCA occlusion and conservative treatment.

The study protocol was reviewed and approved by the Institutional Review Board of the Catholic University, College of Medicine (Registry No. VC20RISI0161), which waived the requirement for informed consent.

### 3.2. Endovascular Intervention Protocols

Endovascular procedures were performed by three neuro-interventionists. EVT involved thrombus retraction with a 6-mm retrievable stent, such as a Solitaire FR (ev3/Covidien, Irvine, CA, USA) or Trevo XP ProVue (Stryker Neurovascular, Fremont, CA, USA) [17,18]. A balloon-guided catheter (Merci, Concentric Medical, Mountain View, CA, USA; or FlowGate2, Stryker Neurovascular) was used in some instances. In other instances, an additional intermediate catheter, the 5F SOFIA (MicroVention Terumo, Tustin, CA, USA) or 6F AXS Catalyst 6 (Stryker Neurovascular) was used for the Solumbra technique. The device or method for endovascular procedures were selected by neurosurgeon’s decisions based on patients’ clinical and vascular conditions, but not performed exclusively for a particular purpose.

### 3.3. Assessment

Clinical assessments were conducted at 2 weeks (12–16 days), 4 weeks (26–30 days), and 6 months (180–210 days) after onset. Functional disability was assessed using the modified Barthel Index (MBI), which consists of 10 subscales with scores ranging from 0 to 100 regarding the basic activities of daily living (ADL) as the primary outcome [19,20,21], the mRS (0–6), functional ambulation category (FAC, 0–5), and dysphagia outcome severity scale (DOSS, 1–7) scores were assessed as other functional measures of disability, handicap, gait, and eating [7,22,23].

### 3.4. Statistical Analysis

Statistical analyses were performed using IBM SPSS Statistics, version 21.0 (IBM Corp., Armonk, NY, USA). The patients’ demographic and clinical characteristics were compared using the chi-squared test for categorical variables and Mann–Whitney U test for continuous variables. Two-factor (EVT × time) repeated measures analysis of variance was performed to determine the effects of EVT on MBI, FAC, mRS, and DOSS scores. All tests were two-tailed and *p*-values ≤ 0.05 were considered statistically significant.

## 4. Results

Table 1 presents the patients’ demographic and clinical characteristics. The distributions of age, sex, sides of stroke did not differ between two groups. The mRS, MBI, FAC, and DOSS for two groups up to 6 months were presented in Table 2.

**Figure 1:**
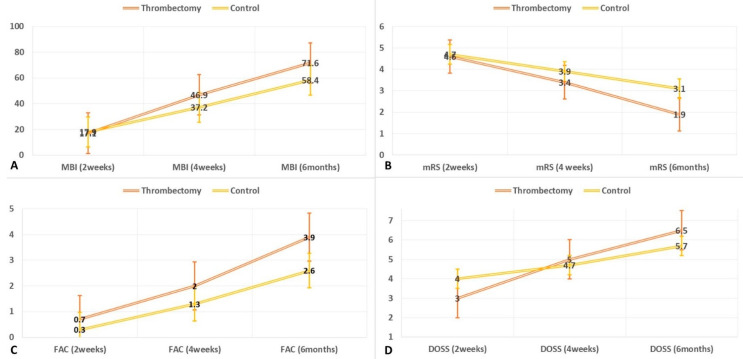
Functional recovery after thrombectomy. The MBI, mRS, FAC, and DOSS scores changed significantly according to group and time. The graph indicates the changes of the activities of daily living ((**A**); modified Barthel Index, MBI, 0–100), handicap ((**B**); modified Rankin Scale, mRS, 0–6), gait ((**C**); functional ambulation category, FAC, 0–5), and eating ((**D**); dysphagia outcome severity scale, DOSS, 1–7). The numbers represent mean values of each groups for each times, and bar lines represent standard deviation of each groups for each times.

The thrombectomy group showed significant improvements in MBI scores with time, compared to the control: values for two weeks, four weeks, six months; 17.1, 46.9, 71.6 for thrombectomy group, 17.9, 37.5, 59.1 for control (all *p* < 0.05, Table 2, Figure 1).

The thrombectomy group showed significant improvements in mRS with time, compared to the control: values for two weeks, four weeks, six months; 4.6, 3.4, 1.9 for thrombectomy, 4.7, 3.9, 3.1 for control (all *p* < 0.05, Table 2, Figure 1). The thrombectomy group showed significant improvements in FAC values with time, compared to the control: values for two weeks, four weeks, six months; 0.7, 2.0, 3.9 for thrombectomy, 0.3, 1.3, 2.6 for control (all *p* < 0.05, Table 2, Figure 1). The thrombectomy group showed significant improvements in DOSS values with time, compared to the control: values for two weeks, four weeks, six months; 3.0, 5.0, 6.5 for thrombectomy, 4.0, 4.7, 5.7 for control (all *p* < 0.05, Table 2, Figure 1).

## 5. Discussion

The use of EVT has resulted in high rates of favorable outcomes worldwide. The outcome and recovery are important considerations for clinicians who treat patients with stroke. Most research regarding EVT has established the primary outcomes as the mRS score at three months after onset and the overall mortality, where a favorable outcome is an mRS score < 3. Thus far, no study has assessed the long-term functional outcomes of patients with an initial severity mRS score ≥ 3, caused by stroke. We demonstrated that EVT favorably affects the disability, handicap, gait, and eating function up to six months after onset in patients with MCA infarct who exhibited even moderate to severe disability [10,11,15].

This improvement is more than a simple score; it represents a change in functional status for affected patients. In terms of MBI score, the control average of 58 means that patients “should require assistance,” while the mean of 71 with EVT indicates that patients “require minimal assistance or supervision.” The respective scores of the EVT and control groups were 1.9 and 3.1 for the mRS, 3.9 and 2.6 for the FAC, and 6.5 and 5.7 for the DOSS. Based on these results, patients in the control group typically required assistance with routine ADL, did not walk independently (despite the use of a walking device), and consumed modified food (e.g., thickened liquids). In contrast, patients in the EVT group could perform routine ADL with modification, supervision, or minimal assistance; walk independently for some distance with a walking device or orthosis; and consume an unmodified diet. Therefore, we recommend EVT for all patients with acute MCA infarction, including those with severe disability at the initial assessment.

Our study had two limitations: its retrospective nature and small sample size. We conducted a retrospective longitudinal study (up to six months after stroke onset) and used narrow inclusion criteria to achieve two homogeneous groups. In addition, according to recent studies, the gender issue, i.e., that the female sex would worsen the outcome of stroke, has emerged as an important issue for recovery and predicting long-term outcomes for stroke [24,25,26,27]. Moreover, age, particularly aged 70 years or older, reported a negative impact on the 30-month outcomes of stroke [16]. Thus, a large-scale prospective longitudinal study for EVT with several clinical factors on the functional outcome would be needed to address the remaining questions.

## 6. Conclusions

The EVT reduces both disability and handicap, while increasing walking and eating abilities up to six months after onset, in patients with MCA infarct who exhibit moderate to severe disability (initial severity mRS score ≥ 3) from severe stroke.

## Figures and Tables

**Table 1:** Patient demographic and clinical characteristics.

Characteristic	Thrombectomy (*n* = 15)	Control (*n* = 30)	*p* Values
Sex, M/F (n)	10/5	17/13	0.519
Age, (mean ± SD, (range))	64.9 ± 12.3 (45–85)	61.7 ± 8.6 (46–80)	0.262
Side of stroke, R/L (n)	9/6	14/16	0.399
Predisposing disease (%)			
High blood pressure	66.7	63.3	
Diabetes	26.7	30	
Arterial Fibrillation	46.7	23.3	
Dyslipidemia	60	46.7	

n: number, SD: standard deviation. *p*-values were tested using Pearson’s chi-square test for sex and side of stroke, the Mann-Whitney U-test for age.

**Table 2:** Functional recovery with time and thrombectomy.

		Thrombectomy (*n* = 15)	Control (*n* = 30)	*p* Value (Time)	*p* Value (Time*Group)
Modified Rankin Scale (0–6)	2 weeks	4.6 ± 0.6	4.7 ± 0.5	<0.001 ^1^	<0.001 ^1^
	4 weeks	3.4 ± 1.0	3.9 ± 0.8		
	6 months	1.9 ± 0.8	3.1 ± 1.0		
Modified Barthel Index (0–100)	2 weeks	17.1 ± 22.5	17.9 ± 19.1	<0.001 ^2^	0.037 ^2^
	4 weeks	46.9 ± 25.2	37.5 ± 20.7		
	6 months	71.6 ± 19.6	59.1 ± 21.2		
Functional Ambulation Category (0–5)	2 weeks	0.7 ± 1.0	0.3 ± 0.8	<0.001 ^1^	0.009 ^1^
	4 weeks	2.0 ± 1.3	1.3 ± 1.2		
	6 months	3.9 ± 1.0	2.6 ± 1.4		
Dysphagia Outcome Severity Scale (1–7)	2 weeks	3.0 ± 1.0	4.0 ± 1.4	<0.001 ^2^	<0.001 ^2^
	4 weeks	5.0 ± 1.5	4.7 ± 1.1		
	6 months	6.5 ± 0.8	5.7 ± 1.0		

^1^: Mauchly’s test of sphericity, ^2^: Greenhouse-Geisser’s test.

## Data Availability

The data presented in this study are available on request from the corresponding author.

## References

[1] Dombovy M.L., Basford J.R., Whisnant J.P., Bergstralh E.J. (1987). Disability and use of rehabilitation services following stroke in Rochester, Minnesota, 1975–1979. Stroke.

[2] Kwakkel G., van Peppen R., Wagenaar R.C., Dauphinee S.W., Richards C., Ashburn A., Miller K., Lincoln N., Partridge C., Wellwood I. (2004). Effects of Augmented Exercise Therapy Time after Stroke: A Meta-Analysis. Stroke.

[3] Lee K.B., Kim J.S., Hong B.Y., Lim S.H. (2017). Clinical recovery from stroke lesions and related outcomes. J. Clin. Neurosci..

[4] Mundiyanapurath S., Möhlenbruch M., Ringleb P.A., Bösel J., Wick W., Bendszus M., Radbruch A. (2015). Posterior Circulation Acute Stroke Prognosis Early Computed Tomography Score Using Hypointense Vessels on Susceptibility Weighted Imaging Independently Predicts Outcome in Patients with Basilar Artery Occlusion. PLoS ONE.

[5] Zheng T., Zhu X., Liang H., Huang H., Yang J., Wang S. (2015). Impact of early enteral nutrition on short term prognosis after acute stroke. J. Clin. Neurosci..

[6] Jang S.H., Chang C.H., Lee J., Kim C.S., Seo J.P., Yeo S.S. (2013). Functional Role of the Corticoreticular Pathway in Chronic Stroke Patients. Stroke.

[7] Lee K.B., Kim J.S., Hong B.Y., Sul B., Song S., Sung W.J., Hwang B.Y., Lim S.H. (2017). Brain lesions affecting gait recovery in stroke patients. Brain Behav..

[8] Sul B., Lee K.B., Hong B.Y., Kim J.S., Kim J., Hwang W.S., Lim S.H. (2019). Association of Lesion Location With Long-Term Recovery in Post-stroke Aphasia and Language Deficits. Front. Neurol..

[9] Yoo Y.J., Kim J.W., Kim J.S., Hong B.Y., Lee K.B., Lim S.H. (2019). Corticospinal Tract Integrity and Long-Term Hand Function Prognosis in Patients With Stroke. Front. Neurol..

[10] Jovin T.G., Chamorro A., Cobo E., De Miquel M.A., Molina C.A., Rovira A., Román L.S., Serena J., Abilleira S., Ribó M. (2015). Thrombectomy within 8 Hours after Symptom Onset in Ischemic Stroke. N. Engl. J. Med..

[11] Nogueira R.G., Jadhav A.P., Haussen D.C., Bonafe A., Budzik R.F., Bhuva P., Yavagal D.R., Ribo M., Cognard C., Hanel R.A. (2018). Thrombectomy 6 to 24 Hours after Stroke with a Mismatch between Deficit and Infarct. N. Engl. J. Med..

[12] Yi H.J., Sung J.H., Lee M.H., Lee N.H. (2019). Experience of the New FlowGate2 Device as a Balloon Guide Catheter for Ischemic Stroke Intervention. World Neurosurg..

[13] Yi H.J., Lee D.H., Sung J.H. (2020). Comparison of FlowGate2 and Merci as balloon guide catheters used in mechanical thrombectomies for stroke intervention. Exp. Ther. Med..

[14] Goyal M., Demchuk A.M., Menon B.K., Eesa M., Rempel J.L., Thornton J., Roy D., Jovin T.G., Willinsky R.A., Sapkota B.L. (2015). Randomized Assessment of Rapid Endovascular Treatment of Ischemic Stroke. N. Engl. J. Med..

[15] Wollenweber F.A., Tiedt S., Alegiani A., Alber B., Bangard C., Berrouschot J., Bode F.J., Boeckh-Behrens T., Bohner G., Bormann A. (2019). Functional Outcome Following Stroke Thrombectomy in Clinical Practice. Stroke.

[16] Yoo J., Hong B., Jo L., Kim J.-S., Park J., Shin B., Lim S. (2020). Effects of Age on Long-Term Functional Recovery in Patients with Stroke. Medicina.

[17] Kim J., Yi H.J., Lee D.H., Sung J.H. (2019). Safety and Feasibility of Using Argatroban Immediately After Mechanical Thrombectomy for Large Artery Occlusion. World Neurosurg..

[18] Yi H.J., Lee D.H., Kim S.U. (2018). Effectiveness of Trevo stent retriever in acute ischemic stroke: Comparison with Solitaire stent. Medicine.

[19] Kim G., Min D., Lee E.-O., Kang E.K. (2016). Impact of Co-occurring Dysarthria and Aphasia on Functional Recovery in Post-stroke Patients. Ann. Rehabil. Med..

[20] Kim S.C., Shin Y.-I., Ko S.H., Kim D.Y., Lee J., Sohn M.K., Lee S.-G., Oh G.-J., Lee Y.-S., Joo M.C. (2020). Factors Associated to Returning Home in the First Year after Stroke. Brain Neurorehabilit..

[21] Nam K.E., Jo L., Jun S.Y., Sung W.J., Kim J.S., Hong B.Y., Sul B., Lim S.H. (2018). Long-term effect of repetitive transcranial magnetic stimulation on disability in patients with stroke. J. Clin. Neurosci..

[22] Van Swieten J.C., Koudstaal P.J., Visser M.C., Schouten H.J., Van Gijn J. (1988). Interobserver agreement for the assessment of handicap in stroke patients. Stroke.

[23] O’Neil K.H., Purdy M., Falk J., Gallo L. (1999). The Dysphagia Outcome and Severity Scale. Dysphagia.

[24] SantaLucia P., Pezzella F., Sessa M., Monaco S., Torgano G., Anticoli S., Zanoli E., Baronello M.M., Paciaroni M., Caso V. (2013). Sex differences in clinical presentation, severity and outcome of stroke: Results from a hospital-based registry. Eur. J. Intern. Med..

[25] Viticchi G., Falsetti L., Plutino A., Bartolini M., Buratti L., Silvestrini M. (2020). Sex influence in ischemic stroke severity and outcome among metabolically unhealthy overweight patients. J. Neurol. Sci..

[26] Tomita H., Hagii J., Metoki N., Saito S., Shiroto H., Hitomi H., Kamada T., Seino S., Takahashi K., Baba Y. (2015). Impact of Sex Difference on Severity and Functional Outcome in Patients with Cardioembolic Stroke. J. Stroke Cerebrovasc. Dis..

[27] Renoux C., Coulombe J., Li L., Ganesh A., Silver L., Rothwell P.M. (2017). Confounding by Pre-Morbid Functional Status in Studies of Apparent Sex Differences in Severity and Outcome of Stroke. Stroke.

